# Clustering of countries based on dairy productivity characteristics of Holstein cattle for breeding material selection

**DOI:** 10.14202/vetworld.2024.1108-1118

**Published:** 2024-05-17

**Authors:** A. F. Petrov, O. V. Bogdanova, K. N. Narozhnykh, E. V. Kamaldinov, K. S. Shatokhin, V. V. Gart, S. G. Kulikova, T. A. Zhigulin

**Affiliations:** Department of Veterinary Genetics and Biotechnology, Novosibirsk State Agrarian University, Novosibirsk, 630039, Russia

**Keywords:** breeding material, cattle productivity, dairy productivity traits, Holstein cattle

## Abstract

**Background and Aim:**

The aim of any breeding process is to create a herd based on certain parameters that reflect an ideal animal vision. Targeted herding involves selecting the source of breeding material to be imported from another country. Therefore, there is a problem in selecting a breeding material importer to rapidly form a uterine canopy with the required properties. The purpose of this study was to evaluate a set of predictive milk productivity traits in Holstein cattle across countries.

**Materials and Methods:**

This research was based on records of 819,358 recorded animals from 28 countries born after January 1, 2018, from open databases. We used the Euclidean metric to construct dendrograms characterizing the similarity of countries according to the complex milk productivity traits of the daughters of bulls. The Ward method was used to minimize intracluster variance when forming clusters and constructing the corresponding diagrams. Principal component analysis was used to reduce dimensionality and eliminate the effect of multicollinearity. The principal components were selected using the Kaiser–Harris criteria.

**Results:**

A ranking of multidimensional complex milk productivity traits in different countries over the past 5 years was performed. A group of leading countries led by the USA was established according to the studied indicators, and the possible reasons for such a division into groups were described.

**Conclusion:**

The pressure of purposeful artificial selection prevails in comparison with the pressure of natural selection concerning milk productivity traits in a certain group of countries, which allows specialists to choose suppliers when buying breeding animals and materials. The findings are based solely on data from recorded animals, which may not represent the entire breed population within each country, especially in regions where record-keeping may be inconsistent. It is expected that further studies will include regional data from large enterprises not part of Interbull, with mandatory verification and validation. An important element of such work is seen as the ability to compare the milk productivity of populations from different countries using a different scale, as well as studying the differentiation of countries by other selection traits of dairy

## Introduction

In today’s world, one of the most pressing issues is to provide a sufficient quantity of food with high nutritional value to the population. Throughout human history, milk and dairy products have been a traditional source of this kind of food. Many publications justify the benefits of consuming natural cow’s milk for human nutrition [1–3]. The aim of any breeding process is to create a herd with certain parameters that reflect the vision of the ideal animal. Under optimal environmental conditions, it is possible to observe the maximum release of genetic potential in relation to selectable traits. Targeted herding involves selecting the source of breeding material to be imported from a country. Each country that has developed animal husbandry has its own breeding index, which affects its population. Each herd shall be established to obtain dairy raw material and maximize the profit. The solution to this problem is closely linked to the mathematical apparatus used and the resulting breeding indices, which are able to distinguish between populations from different countries with different climatic and economic conditions in which targeted selection is carried out. The solution to this problem is inextricably linked to dairy cattle breeding and the genetic potential of bred animals, the realization of which depends on natural and climatic characteristics and production culture [4–7]. The effectiveness of improving genetic potential is directly linked to the state of the breeding stock and the breeding material used. There are differences in the methods and methods used to determine the breeding value of livestock under changing production and climate conditions. This often leads to a shortage of raw materials for dairy products and, as a result, milk products. To neutralize these consequences, it is necessary to do the following: Constant monitoring of population and subpopulation estimates around the world enables us to rank countries according to economically useful traits and accelerate genetic progress [8–10].

Assessing the genetic potential under specific environmental conditions is often associated with applied mathematical approaches and models [11–13]. Since the 1990s, mixed models and an index approach have been traditionally used for livestock breeding [14, 15]. The main advantage of this approach is the complexity of the assessment of the breeding characteristics of the animal, which is almost impossible to achieve if each of the selected characteristics is separately selected [16]. It is considered that the introduction of breeding indices allowed the observed genetic progress of dairy cattle. Unilateral selection in terms of milk productivity contributes to significant progress according to the selected criterion but increases the risk of animal degradation due to other characteristics. Modern breeding minimizes these risks by incorporating animal viability and fertility assessment in the index [17, 18]. The experience of Scandinavian countries (in particular Sweden) shows that there is a possibility of an increase in the milk production of cows without a significant reduction in reproductive characteristics.

The structure of a particular index often depends on the purpose of breeding on a particular farm, region or country. The index used in one region does not always agree with the selection vector of another region [19–24]. For example, in Australia, Denmark, and the United Kingdom, an increase in milk productivity is used in the national indices but not in Canada, Switzerland, and Germany [25].

However, it appears reasonable to use data scaling in comparative studies within the framework of a single selection index and to allow an objective assessment of the variability of selection characteristics in the livestock sector. The lifetime performance index (LPI) and total performance index (TPI) are the most common indices that have proven their reliability and relevance in the real sector of the economy [25–27]. At present, animal populations are often compared using genetic similarity indicators. On the one hand, this approach makes it possible to consider the genetic structure. On the other hand, the connection between genetic markers and some quantitative traits under different production conditions has not always been considered [28–31]. The use of phenotypic distances makes it possible to solve this problem and reflects the final result of the genotype-environment interaction [32–36].

To fully understand the level of milk productivity of livestock, depending on the country of origin, several indicators should be included, including milk yield in kg, milk fat and protein content in kg, and milk fat and protein content in percentages. Therefore, this study aimed to assess countries using a set of predictive milk productivity traits in Holstein cattle.

## Materials and Methods

### Ethical approval

This study did not involve any direct intervention with live animals; it exclusively used publicly available data records from the Canadian Dairy Network (CDN) international database. The study adhered to all relevant guidelines for the use of secondary data and respected the privacy and integrity of the data source.

### Study period and location

The research was conducted from October 2022 to May 2023 at the Laboratory of Applied Bioinformatics and the Department of Veterinary Genetics and Biotechnology of the Novosibirsk State Agrarian University.

### Initial data

Initial data for the study were obtained from the Canadian Dairy Network (CDN) international database. As a primary set, summary tables for bulls and cows of the Holstein breed for April 2022 were used. The number of animals was determined on the basis of the number of animal records in the international CDN database that was born after January 01, 2018 (5 years) from April 2022.

Data in the .csv format were sorted into PHP arrays, verified, and recorded in a previously prepared MySQL database management system database. Each animal has been uniquely identified and compared with its country of origin. Information about productivity indicators, general data about animals (number, name, date of birth), and country of origin were allocated in separate tables in the database architecture and interconnected in relationships using external keys. When writing the source array to the database, animals with incomplete or missing information on origin or productivity indicators received an additional flag indicating that such animals could not be used in the study. All reliably confirmed animal records with a complete set of data were extracted and sorted according to the country of origin using SQL. The sets were rewritten in.json and passed to the working environment of the R language v. 4.2.2 (https://r-for-windows.updatestar.com/).

Data presented in this study are publicly available in the CDN database (https://www.cdn.ca/query/individual.php).

The objective of this study was to determine milk productivity traits in Holstein cattle populations from different countries. The objective of this study was to determine the estimated breed values (EBVs) of milk yield (milk EBV, kg), milk fat (fat EBV, kg; fat EBV, %), and protein (protein EBV, kg, protein EBV, %) for 132186 bulls born no earlier than January 1, 2018.

### Formation of a pool of countries for this study

The countries were selected according to the number of evaluated animals with a lower threshold value of 50 individuals. This condition is met by 28 countries (Table-1).

**Table-1:** Pools of countries included in the study.

Country	Animals with a lower threshold value of 50
NZL	1262
CHE	3369
FIN	58
SWE	80
AUS	1159
IRL	306
URY	61
BLR	79
RUS	182
LTU	206
CAN	12246
GBR	1563
ESP	180
POL	55
BRA	415
ARG	646
DNK	398
CZE	298
DEU	6173
JPN	637
USA	88452
FRA	1462
BEL	231
ITA	7019
LUX	60
HUN	138
CHN	1298
NLD	4153

NZL=New Zealand, CHE=Switzerland, FIN=Finland, SWE=Sweden, AUS=Australia, IRL=Ireland, URY=Uruguay, BLR=Belarus, RUS=Russia, LTU=Lithuania, CAN=Canada, GBR=Great Britain, ESP=Spain, POL=Poland, BRA=Brazil, ARG=Argentina, DNK=Denmark, CZE=Czech Republic, DEU=Germany, JPN=Japan, USA=USA, FRA=France, BEL=Belgium, ITA=Italy, LUX=Luxembourg, HUN=Hungary, CHN=China, NLD=The Netherlands

### Statistical analysis

The initial data arrays were analyzed using the statistical programming R language v. 4.2.2 (https://r-for-windows.updatestar.com/) and RStudio IDE v. 2022.12.0 (https://dailies.rstudio.com/version/2022.12.0+353/).

The Euclidean metric was used to construct dendrograms characterizing the similarity of countries according to the complex features of the milk productivity of the daughters of bulls [37, 38]. The Ward method was used to minimize intracluster variances when forming clusters and constructing the corresponding diagrams [38, 39].

The principal component method was used to reduce the dimensionality and eliminate the effect of multicollinearity [40, 41]. Before the analysis, the initial data were standardized. The principal components were selected using the Kaiser–Harris criterion, according to which components with eigenvalues exceeding one should be used, and by the scree plot, where the eigenvalues are depicted with the component numbers [42, 43]. A bend is usually visible on such a graph, highlighting the principal components [44, 45].

The study involved the calculation of ranks according to the predicted LPI milk productivity traits for cows from each of the 28 countries for which records in the CDN database for the past 5 years were available (Table-2). We compared the indicators of milk yield (kg), fat (kg, %), and protein (kg, %)

**Table 2:** Ranks of countries based on milk productivity.

Country	Milk yield	Fat (kg)	Protein (kg)	Fat (%)	Protein (%)	Average rank	SS*
USA	4	1	3	4	6.5	3.7	2.8
NLD	6	6	4	8	5	5.8	2.2
GBR	17	2	10	2	3	6.8	10.2
CHN	9	3	7	5	11	7.0	4.0
BEL	5	4	7	9	11	7.2	3.8
CZE	1	5	2	13.5	16	7.5	8.5
DEU	2	9	1	21	8	8.2	12.8
JPN	3	7	7	15	16	9.6	6.4
HUN	10	10	7	17.5	6.5	10.2	7.3
POL	12	8	14	6.5	11	10.3	3.7
DNK	14	17	7	20	4	12.4	7.6
FRA	11	13	12.5	17.5	11	13.0	4.5
ARG	15	11	15.5	11	21.5	14.8	6.7
LTU	19	13	20	6.5	16	14.9	5.1
AUS	20	15	19	10	11	15.0	5.0
ITA	8	17	12.5	22.5	16	15.2	7.3
LUX	7	19	11	25	20	16.4	8.6
NZL	27	27	27	1	1	16.6	10.4
BRA	13	13	15.5	19	23	16.7	6.3
CAN	18	17	17.5	13.5	21.5	17.5	4.0
IRL	28	28	28	3	2	17.8	10.2
ESP	16	20	17.5	27	24.5	21.0	6.0
SWE	24	21	24	12	24.5	21.1	3.4
FIN	25	25	22	24	16	22.4	2.6
CHE	26	26	26	16	19	22.6	3.4
URY	23	22	24	22.5	26.5	23.6	2.9
BLR	22	23	21	28	26.5	24.1	3.9
RUS	21	24	24	26	28	24.6	3.4

Note:

* SS is the average sum of rank deviations. NZL=New Zealand, CHE=Switzerland, FIN=Finland, SWE=Sweden, AUS=Australia, IRL=Ireland, URY=Uruguay, BLR=Belarus, RUS=Russia, LTU=Lithuania, CAN=Canada, GBR=Great Britain, ESP=Spain, POL=Poland, BRA=Brazil, ARG=Argentina, DNK=Denmark, CZE=Czech Republic, DEU=Germany, JPN=Japan, USA=USA, FRA=France, BEL=Belgium, ITA=Italy, LUX=Luxembourg, HUN=Hungary, CHN=China, NLD=The Netherlands


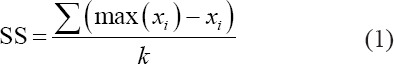


where:

max(x_i_) is the maximum value of the rank of the studied traits calculated for a particular country;

x_i_ is the value of the rank of an arbitrary trait in a particular country;

k is the number of traits.

## Results

By comparing the ranks of the countries according to the studied characteristics, it is possible to determine how the selection vector in a particular country is consistent with the selection based on the milk productivity. The United States and the Netherlands, where dairy cows show the greatest genetic potential, occupied the leading position in this list. Russia and Belarus were at the end of the list, which may indicate limited opportunities to exploit the genetic potential of bred cattle. This approach is economically justified and is often linked to the feeding of animals without the need for additional feed additives.

New Zealand and Ireland have the lowest absolute indicators of milk productivity (milk yield, fat, protein [kg]) and the highest relative indicators (fat, protein [%]), indicating a different method of selection, which is linked to the development of pasture cattle breeding with cheap natural resources.

The lowest level of rank variability in the Netherlands can also be considered noteworthy in the list, whereas Germany, on the other hand, was characterized by contrasting levels of milk yield and milk solid content (Table-2).

**Figure-1:**
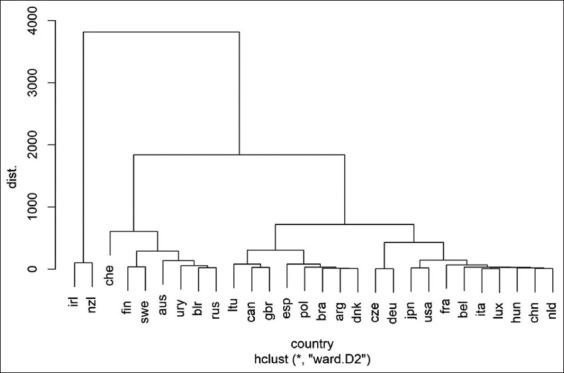
Dendrogram of similarities among countries in terms of the milk productivity of bull daughters. Note: NZL=New Zealand, CHE=Switzerland, FIN=Finland, SWE=Sweden, AUS=Australia, IRL=Ireland, URY=Uruguay, BLR=Belarus, RUS=Russia, LTU=Lithuania, CAN=Canada, GBR=Great Britain, ESP=Spain, POL=Poland, BRA=Brazil, ARG=Argentina, DNK=Denmark, CZE=Czech Republic, DEU=Germany, JPN=Japan, USA=United States, FRA=France, BEL=Belgium, ITA=Italy, LUX=Luxembourg, HUN=Hungary, CHN=China, NLD=The Netherlands.

The country clustering on the dendrogram (Figure-1) allowed us to identify three main groups. The first group included only Ireland and New Zealand. They are located far from other countries due to the low productivity of animals due to climatic conditions and the technology used [46, 47]. Switzerland, Finland, Sweden, Australia, Uruguay, Belarus, and Russia participated in the second group. The remaining 19 countries form the third cluster.

This grouping of countries can be explained mainly by comparing ranks (Table-2) and the differences in cattle housing between New Zealand and Ireland. The only difference is that Australia, which has an average ranking of 15, belongs to the same category as countries with a ranking between 21.1 and 24.6 (the second category). It is also doubtful whether Spain should be classified as a third cluster country since the difference between its middle rank and that of neighboring Sweden in cluster two is 0.1 and that of Ireland (cluster three) is 3.2. This appears to be due to the peculiarities of the distance algorithm and the dimensionality of the other order.

The main component method was used as a way of grouping livestock populations of different countries to arrive at a final conclusion regarding the division of countries into groups.

The most important stage in assessing the applicability of the chosen method is the selection of equations that make it possible to interpret the variability of the complex feature under consideration (Table-3). According to the Kaiser rule [48, 49], when selecting the principal components, it is necessary to pay attention to eigenvalues exceeding one. These results indicate the need to evaluate standardized indicators of milk productivity in two-dimensional space.

**Table 3:** Eigenvalues of the principal components and the percentage of explained variability of features.

Component	Eigenvalue	Variance, %	Cumulative variance, %
1	3.2007	64.0143	64.0143
2	1.5386	30.7726	94.7870
3	0.2543	5.0857	99.8727
4	0.0045	0.0892	99.9618
5	0.0019	0.0382	100.0000

Table-4 shows the contribution of the variable equations to the principal components (in percentage). The division of variables by measurement units was noteworthy. The first component was characterized by traits in absolute terms (kg) with a share of more than 97%, while the second component consisted mainly of traits measured in percentages (more than 90%). The values of variables did not carry any semantic load for the rest of the equations.

**Table 4:** Interpreted variability in milk productivity traits, %.

Variables	Dim. 1	Dim. 2	Dim. 3	Dim. 4	Dim. 5
EBV_milk_kg	30.879	0.535	0.738	9.527	58.322
EBV_fat_kg	23.237	15.567	5.613	55.203	0.380
EBV_protein_kg	26.938	7.317	9.300	18.835	37.610
EBV_fat_percent	6.885	44.954	34.349	13.623	0.189
EBV_protein_percent	12.061	31.627	50.000	2.812	3.500

The most significant contribution of variables in the equations of the principal components exceeded 25% and accounted for features measured in absolute values (component 1) and relative values (component 2). It should be noted that the high correlation coefficients and the determination of individual variables with the values of the functions of the main components confirm the correctness of the conclusions made earlier (Table-5).

**Table-5:** Correlations and coefficients of determination between the traits and the main components.

Trait	Correlations		Determination coefficients	
	
	Dim. 1	Dim. 2	Dim. 1	Dim. 2
EBV_milk_kg	0.9942	0.0907	0.9884	0.0082
EBV_fat_kg	0.8624	0.4894	0.7437	0.2395
EBV_protein_kg	0.9286	0.3355	0.8622	0.1126
EBV_fat_percent	-0.4694	0.8317	0.2204	0.6917
EBV_protein_percent	-0.6213	0.6976	0.3860	0.4866

**Figure-2:**
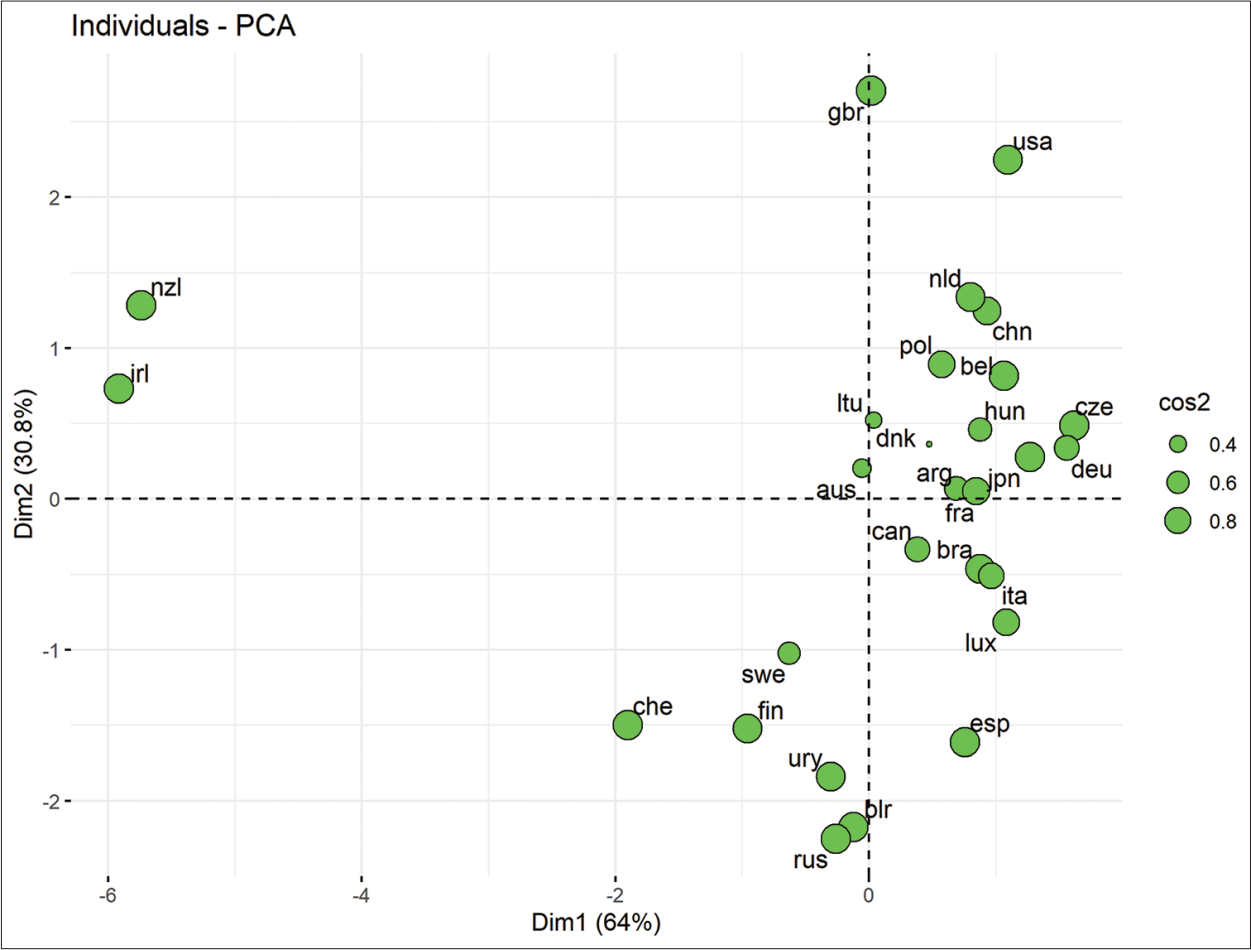
Distribution of countries in the space of principal components (cos2 is the square of correlations of variables with components). Note: NZL=New Zealand, CHE=Switzerland, FIN=Finland, SWE=Sweden, AUS=Australia, IRL=Ireland, URY=Uruguay, BLR=Belarus, RUS=Russia, LTU=Lithuania, CAN=Canada, GBR=Great Britain, ESP=Spain, POL=Poland, BRA=Brazil, ARG=Argentina, DNK=Denmark, CZE=Czech Republic, DEU=Germany, JPN=Japan, USA=United States, FRA=France, BEL=Belgium, ITA=Italy, LUX=Luxembourg, HUN=Hungary, CHN=China, NLD=The Netherlands.

The separation of two components allows countries to be ranked on the basis of milk productivity traits in animals in two-dimensional space (Figure-2).

By analyzing the distribution of countries in the space of the main components, it can be concluded that the studied populations formed four separate groups in the plane. Australia appears to be in the third cluster, which is fully consistent with its position shown in Table-2. Spain has a border position between the second and the third groups. Considering the dendrogram data (Figure-1), this country can be placed into the third group (Figure-2).

The United Kingdom and the United States form a separate (fourth) group of principal components. In these countries, animals showed the highest values for the second principal component (Figure-2), which describes traits in relative units of measurement. The assessed countries were characterized by a high level of indicators, expressed in absolute values (Table-2).

## Discussion

### Comparative analysis of milk productivity traits by country

The similarities between countries such as New Zealand and Ireland are presented in Figure-2. These countries are far from the rest of the countries, which correspond to the first cluster of the dendrogram (Figure-1). These countries had the lowest milk yield, fat, and protein in absolute units of measurement, while the relative proportion of the studied solids was the highest (Table-2).

In contrast to private farms, the dairy industry in Ireland is increasingly being taken over by large multinational companies. The milk production quotas introduced in the European Union in 1984, which limit milk production and provide state subsidies, have had a greater impact on the sector. In 2015, their cancelation was accompanied by an increase in milk production since farmers had to rely only on their efficiency to make a profit. New Zealand borrowed a dairy cattle breeding system from Ireland (keeping animals on pastures) but did not adopt farming as a family business [17]. In Ireland, the pasture period lasts for up to 300 days and for the rest of the year, the cows eat grass silage, which is why milk production is not seasonal. In Ireland, Holstein is the most common breed of cows, with an average productivity of 5,620 kg of milk with a fat content of 4.2% and protein content of 3.6%. Ireland has become one of the world’s leading producers of infant formula due to the high quality of the milk produced [50–53].

New Zealand had the lowest milk yield (kg), fat (kg), and protein (kg), while the relative content of the studied solids was the highest. This can be explained by the fact that year-round pastures continue to use available natural resources [54, 55]. This approach is also economically justified and involves other livestock breeding programs [51, 56–58], which may indicate a high level of population consolidation.

Dairy cattle populations in other countries significantly differed from those listed above. This may indicate that artificial selection exerts a greater pressure on the studied traits. Switzerland, Finland, Sweden, Uruguay, Belarus, and Russia, which corresponds to the second cluster (Figure-1). Sweden, Switzerland, Finland, and Uruguay should be included in this cluster. Switzerland is far from the other countries in the group. The Holstein breed is not dominant in this country, and the principles of dairy farming differ from those of other countries since Switzerland pays particular attention to the use of natural alpine pastures and animal welfare [59]. Since 2014, there has been a voluntary program of grassland-based milk and meat production in Switzerland, according to which producers should abandon the concentrated type of feeding in favor of grasslands [60]. This management of dairy cows does not contribute to high productivity but contributes to improving the welfare of the animals and the quality of the products obtained. According to the Central Institute for Research on Cattle [61], the average yield in Switzerland is more than 9 thousand kg per cow, with 4.1% and 3.3% fat and protein content. In Scandinavian countries and Switzerland as well as Holstein cows, other dairy breeds, such as Ayrshire and Jersey, are common. In terms of population size, they even surpass Holstein cattle.

Finland and Sweden are neighboring countries that produce milk under similar conditions, pursue the same breeding objectives, and use the Nordic total merit (NTM) index [62, 63]. They distinguish themselves from the other countries of their group. Joint genetic breeding for non-tuberculosis (NTM) has been performed in Sweden, Finland, and Denmark since 2008 because these countries pursue common objectives in cattle breeding. This index is based on an assessment of reproductive and adaptive indicators of livestock. This could have affected the low indices of bulls from these countries. This is due to the negative correlation between productivity and adaptive traits of dairy cattle, which, according to some data, is associated with polymorphisms of switch genes [64]. The productivity of Holstein cattle in these countries exceeds 9,000 kg of milk per year, with a fat content of 4.5%–4.6% and a protein content of 3.5%–3.6% [61, 65].

In the past 35 years, Uruguay has experienced dynamic growth in the dairy sector since the industry has shifted from the internal market to the external market. More than 70% of milk is exported in the form of powdered milk, butter, or cheese. Uruguay is one of the world’s leading producers of milk exports. High competitiveness is based on low costs and is justified by a low concentration of livestock and an average level of use of concentrates [66]. The annual productivity per cow is approximately 5,200 kg with a fat content of 3.7% and protein content of 3.3% [63, 67]. Uruguay and Belarus are among the world’s leaders in milk exports due to the large number of animals [61].

Belarus and Russia (Figure-1) have been paired due to the similarity in the management of dairy cattle breeding, as these countries use similar technologies of maintenance and feeding, and their geographical proximity causes similar economic conditions [68]. A notable feature of the results obtained is the position of Russia and Belarus in the two-dimensional space of the main components (Figure-2). In these countries, there is a similarity of breeding stock in all the characteristics considered. The fat (%) and protein (%) contents were the lowest among all the countries represented. For agricultural organizations in Russia and Belarus, the milk yield per cow in 2020 was 6,728 kg and 5,310 kg [59, 69].

In the third cluster (Figure-1), Brazil and Argentina were the closest with regard to the five indicators. Both countries conduct economic activities under similar conditions, focusing on the maintenance of pastures and obtaining milk with minimal investment [6, 70, 71]. In Brazil, 70% of milk comes from pure Holstein cattle and their crossbreeds. Crossing of Holstein cattle with local breeds is conducted here, which could have an impact on the evaluation of bulls observed in this country. One of the problems associated with dairy cattle breeding in Brazil is the adaptation of Holstein cattle to a hot climate [6, 8], which may also be the reason for the low values observed in the assessment of bulls. In the past 40 years, the breeding of dairy cattle in Argentina has repeatedly undergone economic restructuring. During the same period, there were several crises which did not affect the assessment of the bulls [72].

The majority of farms in Australia are family businesses with a small number of employees. In recent years, however, there has been a global trend toward the consolidation of farms, with an average increase in the number of one enterprise from 107 to 272 cows. These factors limit the growth of milk production in Australia. A small number of employees cannot work with a large number of animals. For the dominant Holstein breed, the productivity of cows in this country was approximately 6,500–8,400 kg for 305 days of lactation, with a fat content of approximately 3.7%–3.8% and a protein content of 3.2-3.3% [4].

Among South Asian countries, China and Japan are the leaders in milk production in recent years, and the Chinese government has made significant efforts to develop the dairy industry. The focus has been on improving the quality of the products and the construction of new modern farms. To increase gross milk production, a large number of cattle, mainly Holstein breeds, were imported. Dairy cattle breeding in Japan underwent active development in the 1950s during the economic recovery after World War II. Milk production has become an important part of the economy and agriculture, and the number of cows has quickly reached the level of European countries. The New Zealand model was chosen as an initial model for dairy cattle breeding, but in Japan, it was not very effective. At present, the milk yield per Holstein cow in Japan is approximately 10,000 kg of milk for 305 days of lactation, with a fat content of almost 4% and a protein content of 3.4% [73].

Canada ranks second in the world after the United States in terms of the number of bulls sold by breeding suppliers. This is because of the high number of bulls produced in Canada. In addition, the Canadian LPI includes a number of estimates that are not included in the American TPI [25]. In addition to increasing herd productivity, Canadian milk producers pay considerable attention to animal welfare and animal health standards [72].

When characterizing the placement of the USA and the UK in the fourth cluster (Figure-2), we notice that the very high probability of high ratings of bulls being obtained in the USA is conditioned by the approaches and methods of breeding aimed at obtaining maximum profit from each dairy cow. TPI is one of the main American breeding indices based on this principle [74]. As a result, the milk productivity of Holstein cows in the USA as of 2021 is 28,047 pounds of milk, 1,121 pounds of milk fat, and 877 pounds of protein per year [74]. We believe that this result is a consequence of purposeful selection, which is confirmed by the structure of selection indices and indicates the prevalence of artificial selection pressure on milk productivity traits compared to natural selection pressure [25].

The United States and Canada were in two different clusters, with bulls being selected for cows regardless of the country of origin [13, 75]. It is therefore difficult to accept the partial isolation of American and Canadian populations of Holstein cattle as a reason for the divergence in different clusters. The discrepancy between the USA and Canada in different clusters can, however, be explained by differences in breeding methods, such as systems for assessing livestock by milk productivity. In particular, the Canadian LPI does not imply an assessment of bulls on the basis of an increase in milk yield [50, 76–78]. These findings suggest that the decisive factor in the assessment of the genetic potential of bulls is the economic conditions and breeding requirements of a particular country, region, or farm, and not the origin of the bull. However, it is difficult to say definitively because of the high degree of variation in the contribution of the component heredity share to the milk productivity traits and adaptive qualities of cattle [79]. According to the catalogs of the world’s leading companies selling stud bull seed (AltaGenetics [n = 314], Semex [n = 239], STgenetics [n = 319], and Worldwide Sires [n = 861]), the share of bulls obtained in the USA is more than 50% of the total, which is an indicator of the high breeding value of American animals [74].

The United Kingdom produces more than 8,000 kg of milk per lactation with a fat content of more than 4% and a protein content of approximately 3.2% [61, 80, 81]. Among European countries other than the European Union, the United Kingdom ranks second after Russia in terms of gross milk production with significantly less livestock (10 times less). However, unlike in the United States, there is no significant share of British bulls in the catalogs of the leading seed suppliers. The main reason is the total number of bulls in these countries [25].

### Implications for dairy breeding and strategic insights

In our study, using Holstein rock as an example, we characterized populations of different countries using a set of predictive indicators of dairy productivity. We used three methods to improve objectivity to understand the inadequacy of using only one method: The country distribution according to the calculated ranks (Table-2) was supplemented by population characterization using phenotypic distances (Figure-1). The main component method made it possible to place countries in a two-dimensional coordinate system objectively relative to each other (Figure-2). All of this made it possible to take into account the implementation of the genotype in a specific environment resulting from production conditions that have their own characteristics in each country. This distinguishes our research approach from those based on the construction of genetic distance.

A description of the current relative position of countries (Figure-2) and the reasons for their entry into a cluster can help experts analyze the direction of the breeding vector and selection pressure on the investigated populations of Holstein cattle from the position of the Canadian system – one of the most common systems for estimating the genetic potential of livestock.

The comprehensive graphical and descriptive information that we have provided may help professionals who, when purchasing breeding material, prefer countries whose population corresponds most to the ultimate goal of breeding during the development or improvement of their herd. Dairy productivity indicators were selected as breeding targets in this study to show how different animal populations differ from country to country. It is important to establish a possible link with the selection vectors reflected in the relevant national indices.

The researcher or practitioner often has the task of determining the position of his herd on his productivity not only based on elementary descriptive statistics but also due to the multidimensional nature of the system of selected coordinates (Figure-2). This will allow us to evaluate the conducted breeding work, adjust the productivity improvement vector, and select the most suitable countries for borrowing genetic and technological components based on the similarity of the selection vector and the technological and environmental conditions (environmental conditions) of the constituents.

Notably, selection is an adaptive process that is adjusted when many factors change. The existing distribution of countries in the main component space may change, and this research may become a retrospective monitoring tool that contributes to historical zootechnical science. Nevertheless, we believe that the observed results are fairly stable due to a slight change in national index structures and the consequent increase in the homogeneity of the uterine herd and the greater consolidation of the selection attributes in a given region of the world global market.

## Conclusion

This study allowed us to evaluate a multidimensional set of milk productivity traits expressed in various units of measurement. The results of this study can be summarized as follows:

- We ranked multidimensional complex milk productivity traits in different countries over the past 5 years. Over the past 5 years, the USA has led a group of top-performing countries based on milk productivity traits, demonstrating the impact of purposeful artificial selection over natural selection processes;
- Differentiation of countries by breeding strategies makes it possible to make informed decisions in the selection of breeding material and to optimize genetic progress and operational efficiency under various climatic conditions.

The results obtained will allow breeders to make a conscious decision on the importing country of breeding material. This will accelerate the achievement of breeding objectives without significant time-consuming efforts to consolidate the herd under certain climatic conditions.

## Data Availability

All data generated are available in the article.

## Authors’ Contributions

AFP: Conceptualization, methodology, data analysis, and writing - original draft preparation. OVB: Data collection, analysis, and interpretation. KNN and VVG: Statistical analysis and interpretation of results. EVK: Literature review and validation of data. KSS: Visualization and presentation of results. SGK and TAZ: Review and editing. All authors have read, reviewed, and approved the final manuscript.
